# Total muscle-to-fat ratio influences urinary incontinence in United States adult women: a population-based study

**DOI:** 10.3389/fendo.2024.1309082

**Published:** 2024-03-28

**Authors:** Dongmei Hong, Hui Zhang, Yong Yu, Huijie Qian, Xiya Yu, Lize Xiong

**Affiliations:** Department of Anesthesiology and Perioperative Medicine, Shanghai Key Laboratory of Anesthesiology and Brain Functional Modulation, Clinical Research Center for Anesthesiology and Perioperative Medicine, Translational Research Institute of Brain and Brain-Like Intelligence, Shanghai Fourth People’s Hospital, School of Medicine, Tongji University, Shanghai, China

**Keywords:** total muscle-to-fat ratio, urinary incontinence, logistic regression analysis, predictive model, lymphocyte count

## Abstract

**Purpose:**

This study aims to investigate the relationship between the total muscle-to-fat ratio (tMFR) and female urinary incontinence (UI), determine whether tMFR can serve as a useful index for predicting UI, and identify factors that may influence this relationship.

**Methods:**

We retrospectively analyzed data from 4391 adult women participating in the National Health and Nutrition Examination Survey (NHANES) conducted between 2011 and 2018. The correlation between tMFR and UI was examined using a dose-response curve generated through a restricted cubic spline (RCS) function, LASSO and multivariate logistic regression. Furthermore, predictive models were constructed incorporating factors such as age, race, hypertension, diabetes, cotinine levels, and tMFR. The performance of these predictive models was evaluated using training and test datasets, employing calibration curves, receiver operating characteristic curves, and clinical decision curves. Mediation effects were also analyzed to explore potential relationships between tMFR and female UI.

**Results:**

In a sample of 4391 adult women, 1073 (24.4%) self-reported experiencing UI, while 3318 (75.6%) reported not having UI. Based on the analyses involving LASSO regression and multivariate logistic regression, it was found that tMFR exhibited a negative association with UI (OR = 0.599, 95% CI: 0.497-0.719, P < 0.001). The results from the restricted cubic spline chart indicated a decreasing risk of UI in women as tMFR increased. Furthermore, the model constructed based on logistic regression analysis demonstrated a certain level of accuracy (in the training dataset: area under the curve (AUC) = 0.663; in the test dataset: AUC = 0.662) and clinical applicability. The mediation analysis revealed that the influence of tMFR on the occurrence of UI in women might potentially occur through the blood index lymphocyte count (P = 0.040).

**Conclusion:**

A high tMFR serves as a protective factor against UI in women. Furthermore, lymphocyte might be involved in the relationship between tMFR and female UI.

## Introduction

An individual experiencing urinary incontinence (UI) involuntarily leaks urine for various reasons (1). It is more prevalent in women (2) and has adverse effects on the female population (3), including their careers and family life. UI can be categorized into three types: stress urinary incontinence (SUI), urge urinary incontinence (UUI), and mixed urinary incontinence (MUI) (4). An illustrative instance is SUI, which refers to involuntary urine leakage during physical activities, sneezing, or coughing. UI is linked to age, obesity, and childbirth (5, 6). According to surveys, the incidence of SUI in the female population can be as high as 24.8% (7), significantly impacting social engagements, mental well-being, and sexual function (3, 8), and adding to the economic and medical burdens for patients to a certain extent (9).

As individuals age and their physical activity decreases, sarcopenia becomes more prevalent. Sarcopenia entails both functional and mass loss in skeletal muscles (10). There’s evidence indicating that obesity escalates the risk of urinary incontinence (11), and the body mass index (BMI) stands as the primary measure of obesity. However, BMI fails to identify a substantial portion of individuals with high body fat content (12) as it doesn’t differentiate between abdominal obesity and age-related obesity. More critically, BMI doesn’t distinguish between the relative quantities of muscle and fat. Research indicates that the muscle-to-fat ratio (MFR) can serve as a biomarker for chronic kidney disease (13). While managing weight and achieving weight loss can effectively decrease the frequency and severity of urinary incontinence in women (14), diminishing body fat and augmenting muscle mass are pivotal for UI prevention. Nonetheless, it remains uncertain whether the MFR can be employed as a predictor of UI in women.

No previous study has explored the correlation between the prevalence of UI in women and the total muscle mass divided by the total body fat mass (tMFR), as assessed by dual-energy x-ray absorptiometry (DXA). Hence, we utilized data from participants within the National Health and Nutrition Examination Survey (NHANES) database to investigate whether tMFR could serve as an indirect radiological predictor for the prevalence of UI in women. Our objective is to offer novel insights into female UI prevention strategies.

## Materials and methods

### Study population

NHANES is a research program designed to assess the health and nutritional status of adults and children in the United States. It integrates interviews and physical examinations, with a focus on various populations and health topics. The diseases investigated by NHANES are diverse and encompass various systems. The program primarily relies on health interviews. The research team includes physicians, medical and health technicians, as well as diet and health interviewers. NHANES is structured to facilitate and incentivize participation, and all participants receive compensation and medical outcome reports. All data collected during the survey are held in strict confidentiality. NHANES obtains informed consent and ethical approval from all participants. The NHANES survey utilized a computer-assisted personal interview system conducted by trained medical personnel. Information on age, race, education, and disease status was obtained through a professional home questionnaire. Body measurements were taken during medical examinations at mobile medical screening centers. Blood samples were collected and quality controlled for all participants except for the very young. For more information about the database, please visit the website https://www.cdc.gov/nchs/nhanes.

In this study, we utilized questionnaire and survey data from NHANES for four consecutive periods (2011-2012, 2013-2014, 2015-2016, and 2017-2018) in a retrospective analysis. Throughout this eight-year timeframe, a cumulative total of 39,196 individuals took part in the NHANES survey, out of which 34,765 were excluded from our study. The inclusion and exclusion criteria for this study were: i) exclusion of male participants, 19,308; ii) exclusion of participants missing information on the UI questionnaire, 9,877; iii) exclusion of participants missing information on total body fat weight, 4,916; iv) exclusion of participants missing information on educational level and family income to poverty ratio, 381; and v) exclusion of participants missing information on history of hypertension, history of diabetes mellitus and blood urea nitrogen, 283. **Supplementary Figure 1** illustrates our screening process. Following the screening criteria, a total of 4,391 women were included in this study.

### Definition of the tMFR

In this study, our aim was to investigate whether tMFR could serve as a physical measurement for predicting UI in women. tMFR is computed by dividing the total body muscle mass by the total body fat mass. Both total body muscle mass and total body fat mass can be quantified using DXA. DXA’s quality control measures include equipment calibration and field inspection observations. Site visits by NCHS staff, subject matter experts from collaborating agencies, and component-specific contract technical consultants were done throughout the year to monitor staff performance as part of quality assurance. Periodic retraining sessions were conducted with the MEC staff. The rigorous schedule of quality control scans provided continuous monitoring of machine performance. The expert review procedures helped to ensure that scan analysis was accurate and consistent. In accordance with the NHANES measurement protocol, the exclusion criteria for DXA are as follows: 1) Pregnancy (confirmed through a positive urine pregnancy test and/or self-report); 2) Self-reported history of recent use of radiographic contrast media (such as barium) within the past 7 days; 3) Self-reported weight exceeding 450 lb or height surpassing 1.75 m.

### Assessment of UI

The outcome variable in this study was female UI, diagnosed by the Kidney Conditions-Urology section of the NHANES database. Participants who responded “yes” to any of the following three questions were categorized as having UI: “In the past 12 months, have you experienced any leakage or loss of control of even a small amount of urine during activities such as coughing, lifting, or exercising?”; “In the past 12 months, have you experienced any leakage or loss of control of even a small amount of urine when experiencing an urge or pressure to urinate and were unable to reach a toilet quickly enough?”; “In the past 12 months, have you experienced any leakage or loss of control of even a small amount of urine without engaging in activities such as coughing, lifting, or exercising, or without experiencing an urge to urinate?”.

### Other covariates

The study also included the following confounding factors, including age, race (Spanish white, non-Hispanic black, Mexican United States, other Hispanic, and other), education (less than 9th grade, 9-11th grade, high school graduate/GED or equivalent, some college or AA degree, and college graduate or higher), marital status (married, widowed, divorced, separated, never married, and living with a partner), family income-to-poverty ratio, history of hypertension, history of diabetes mellitus, vigorous leisure activities, moderate leisure activities, blood urea nitrogen, creatinine, and cotinine. Hypertension and diabetes were diagnosed based on tests performed by a professional physician or self-reported history of hypertension/diabetes. “Do you do any vigorous-intensity sports, fitness, or recreational activities that cause a large increase in breathing or heart rate, such as running or basketball for at least 10 minutes continuously?”, “Do you do any moderate-intensity sports, fitness, or recreational activities that cause a small increase in breathing or heart rate, such as brisk walking, bicycling, swimming, or golf for at least 10 minutes continuously? Serum cotinine levels are a measure of the prevalence and extent of tobacco use, and blood concentrations can be used as markers of active smoking and exposure to second-hand smoke.

### Statistical analysis

To further assess the predictive value of tMFR for the risk of UI in women, we first used LASSO regression (15) to screen for relevant variables and then constructed a nomogram (16) predictive model using P < 0.05 indicators in multivariate logistic regression. We randomly selected 50% of the participants to form a training dataset, and receiver operating characteristic (ROC) curves (17), calibration curves, and decision curve analysis (DCA) curves (18) were used to evaluate the nomograms predictive model. The remaining 50% of participants form an internal validation set to validate the model. In addition, mediation effect was applied to explore the internal mechanism between tMFR and female UI.

The variables used in this study include both continuous and categorical variables. The mean ± standard deviation (SD) is used for continuous variables that follow a normal distribution, the median (upper and lower quartiles) is used for continuous variables that do not follow a normal distribution, and proportions are used for categorical variables. In this study, the chi-squared test is used for statistical difference analysis of categorical variables. We used logistic regression to estimate the odds ratio (OR) and 95% confidence interval (CI) between tMFR and the risk of UI in women and visualized using the ‘forester’ package. In addition, dose curves were used to show the dose-response relationship between tMFR and UI in women. Statistical analyses were performed using SPSS (version 24.0) and R (version 4.2.3) software, and graphs were created using R (version 4.2.3) and Adobe Illustrator (version 26.0) software. P<0.05 (two-sided) was considered statistically significant.

## Results

### Participant characteristics

The study included 4391 women who met the criteria in the NHANES database from 2011 to 2018. The baseline clinical characteristics of all participants are shown in **Supplementary Table 1**, with 1073 (24.4%) self-reporting UI and 3318 (75.6%) without UI. We then assessed the clinical characteristics of all women using chi-squared tests, including variables such as age (P < 0.001), race (P < 0.001), education level (P < 0.001), marital status (P < 0.001), household income (P < 0. 001), hypertension (P < 0.001), diabetes mellitus (P < 0.001), vigorous leisure time activity (P < 0.001), moderate leisure time activity (P = 0.002), blood urea nitrogen (P < 0.001), creatinine (P = 0.006), cotinine (P = 0.001) and tMFR (P < 0.001). We found that the average age of UI patients was 46 years, and women who did not engage in leisure activities were more likely to develop UI.

### Relationship between tMFR and female UI

**Figure 1:**
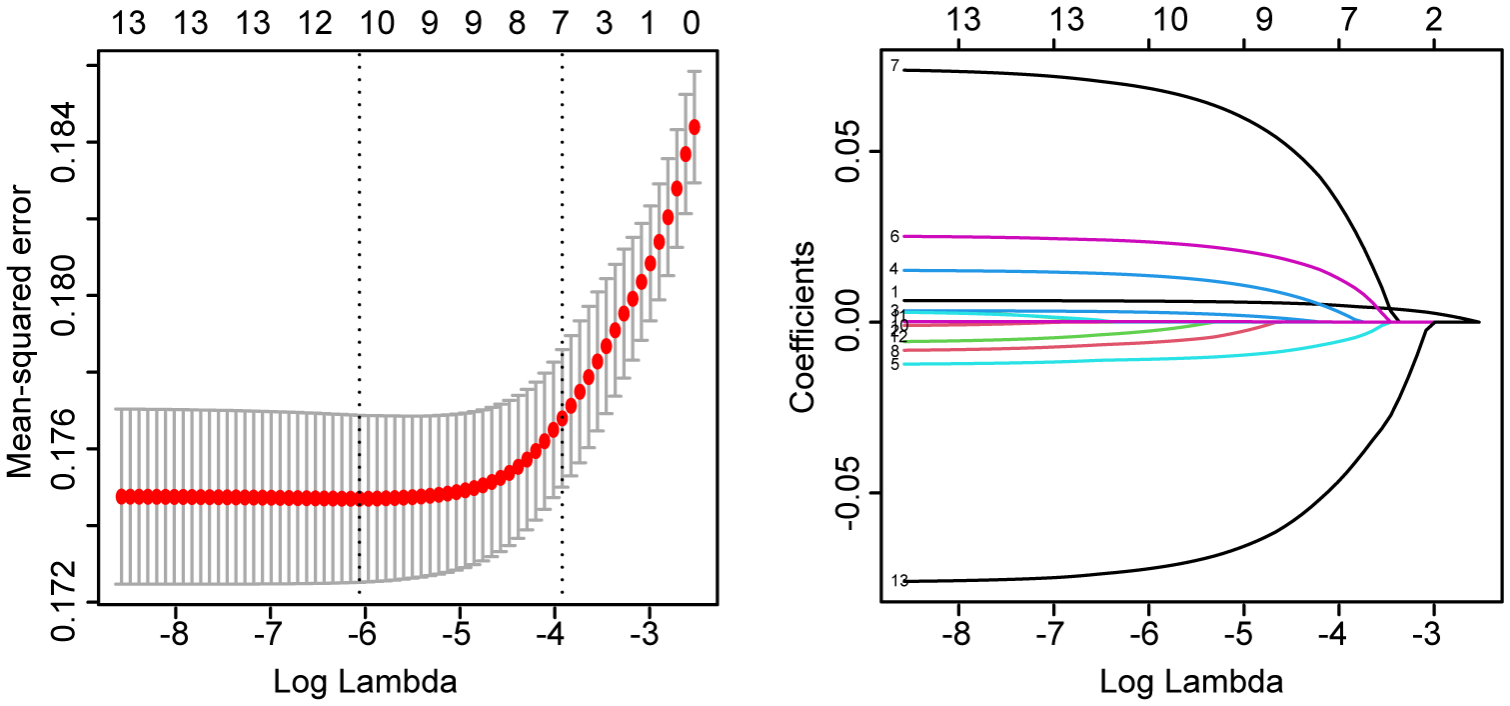
LASSO regression results for age, race, education level, marital status, ratio of family income to poverty, hypertension, diabetes, vigorous recreational activity, moderate recreational activity, blood urea nitrogen, creatinine, cotinine levels, and tMFR versus female UI for all participants.

**Figure 2:**
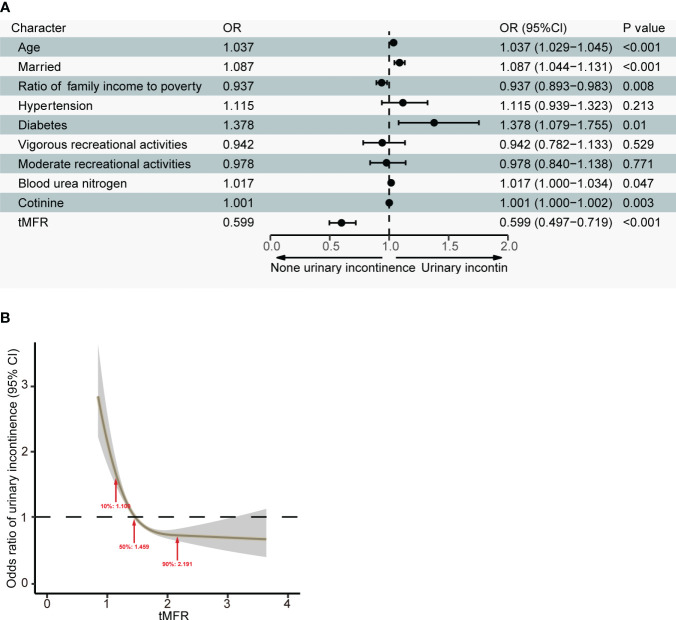
**(A)** Results of a multivariate logistic regression analysis of age, marital status, household income to poverty ratio, hypertension, diabetes, vigorous recreational activity, moderate recreational activity, blood urea nitrogen, cotinine levels, and tMFR in all participants and prevalence of UI in women; **(B)** Dose-response relationship between tMFR and female UI.

We further analyzed the relationship between tMFR and UI. We used LASSO regression to screen 4391 participants for variables. According to **Figure 1**, age, marital status, household income and poverty ratio, hypertension, diabetes, intensive leisure time activity, moderate leisure time activity, blood urea nitrogen, cotinine and tMFR were the variables screened by LASSO regression. Multivariate logistic regression analysis (**Figure 2A**) was performed using the above indicators to obtain age (OR = 1.037, 95%CI = 1.029-1.045, P < 0.001), marital status (OR = 1.087, 95%CI = 1.044-1.131, P < 0.001), household income and poverty ratio (OR= 0.937, 95% CI, 0.893-0.983, P= 0. 008), diabetes mellitus (OR = 1.378, 95%CI = 1.079-1.755, P = 0.010), blood urea nitrogen (OR = 1.017, 95%CI = 1.000-1.034, P = 0.047), cotinine (OR= 1. 001, 95%CI, 1.000-1.002, P= 0.003) and tMFR (OR = 0.599, 95%CI: 0.497-0.719, P < 0.001) were strongly associated with the prevalence of UI in women. In addition, we used the dose curve in the restricted cubic spline (RCS) plot (19) to analyze the dose-response relationship between tMFR and UI in women. **Figure 2B** showed that the risk of UI in women decreased with increasing tMFR, which was inversely related.

### Model development and validation

**Figure 3:**
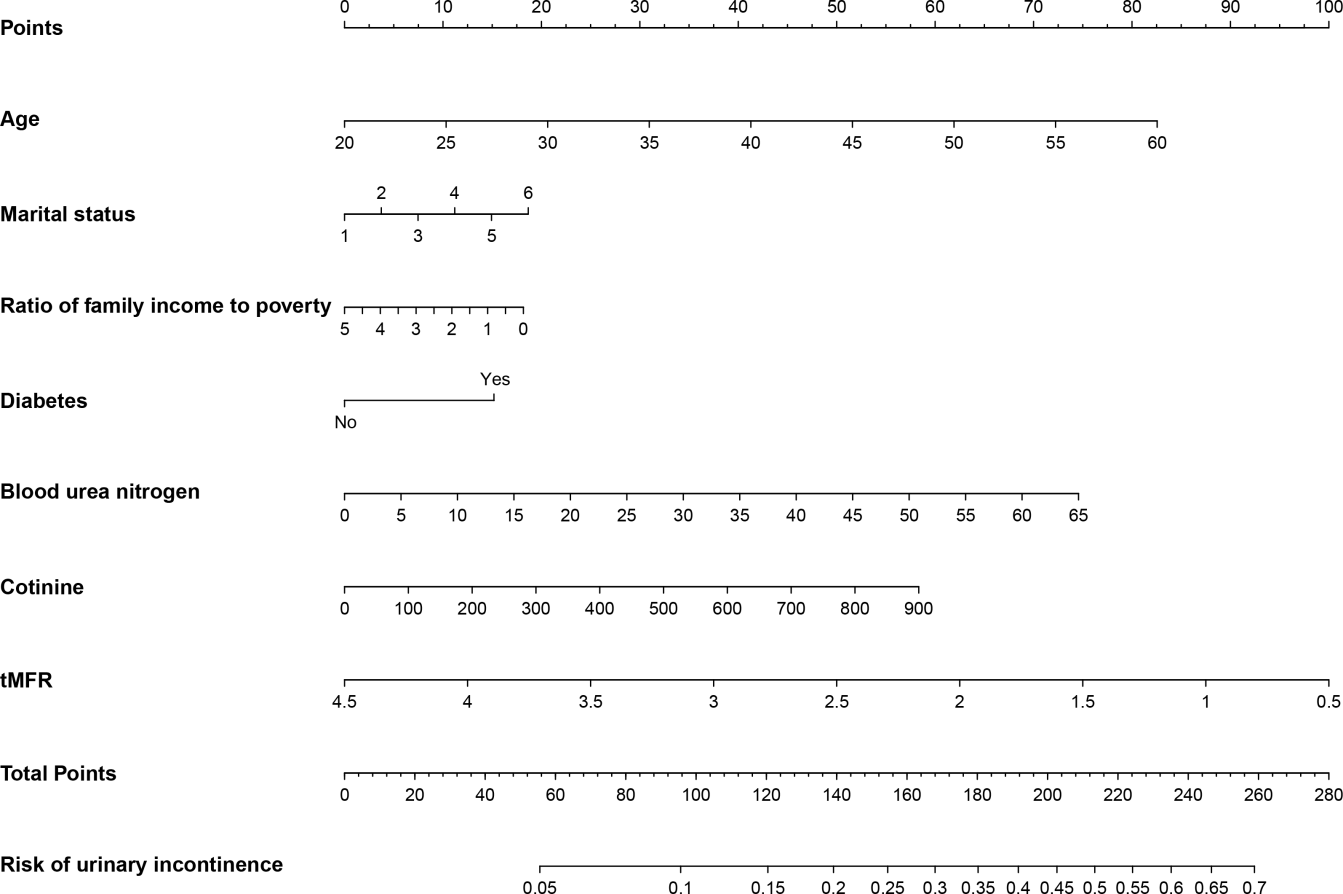
Nongram predicting the risk of UI in women were calculated by scoring each numerical level for age, marital status, household income to poverty ratio, diabetes, blood urea nitrogen, cotinine levels, and tMFR for all participants.

**Figure 4:**
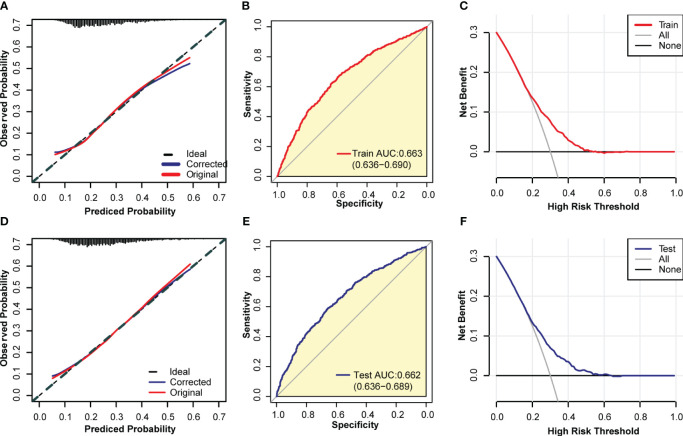
Evaluation of the model. **(A)** a calibration curve of the model constructed from the training set; **(B)** ROC curve of the model constructed from the training set; **(C)** DCA curve of the model constructed from the training set; **(D)** Calibration curve of the model constructed from the internal test set; **(E)** ROC curve of the model constructed from the internal test set; **(F)** DCA curve of the model built from the internal test set.

According to the results of multivariate logistic regression, we randomly selected 50% of the participants as the training dataset and the remaining 50% as the internal test dataset. We constructed a nomogram to predict the risk of UI in women based on the training dataset (**Figure 3**). To verify the predictive performance of the nomogram model in the test dataset, we used the calibration curve as a calibration tool to evaluate the accuracy of the nomogram model. **Figures 4A, D** showed the calibration of the training and test datasets. The predicted risk of UI in women is highly consistent with the actual observed results, indicating that the calibration effect of the nomogram is significant. **Figures 4B, E** showed the ROC results for the training and test datasets, with an area under the curve (AUC) of 0.663 (0.636-0.690) and 0.662 (0.636-0.689), respectively. Those indicated that the constructed model is highly accurate in predicting the UI in women. Finally, we evaluated the clinical value of the nomogram in predicting the risk of UI in women using DCA curves. **Figures 4C, F** showed that patients could benefit from the predicting model.

### Mediation effect analysis

**Figure 5:**
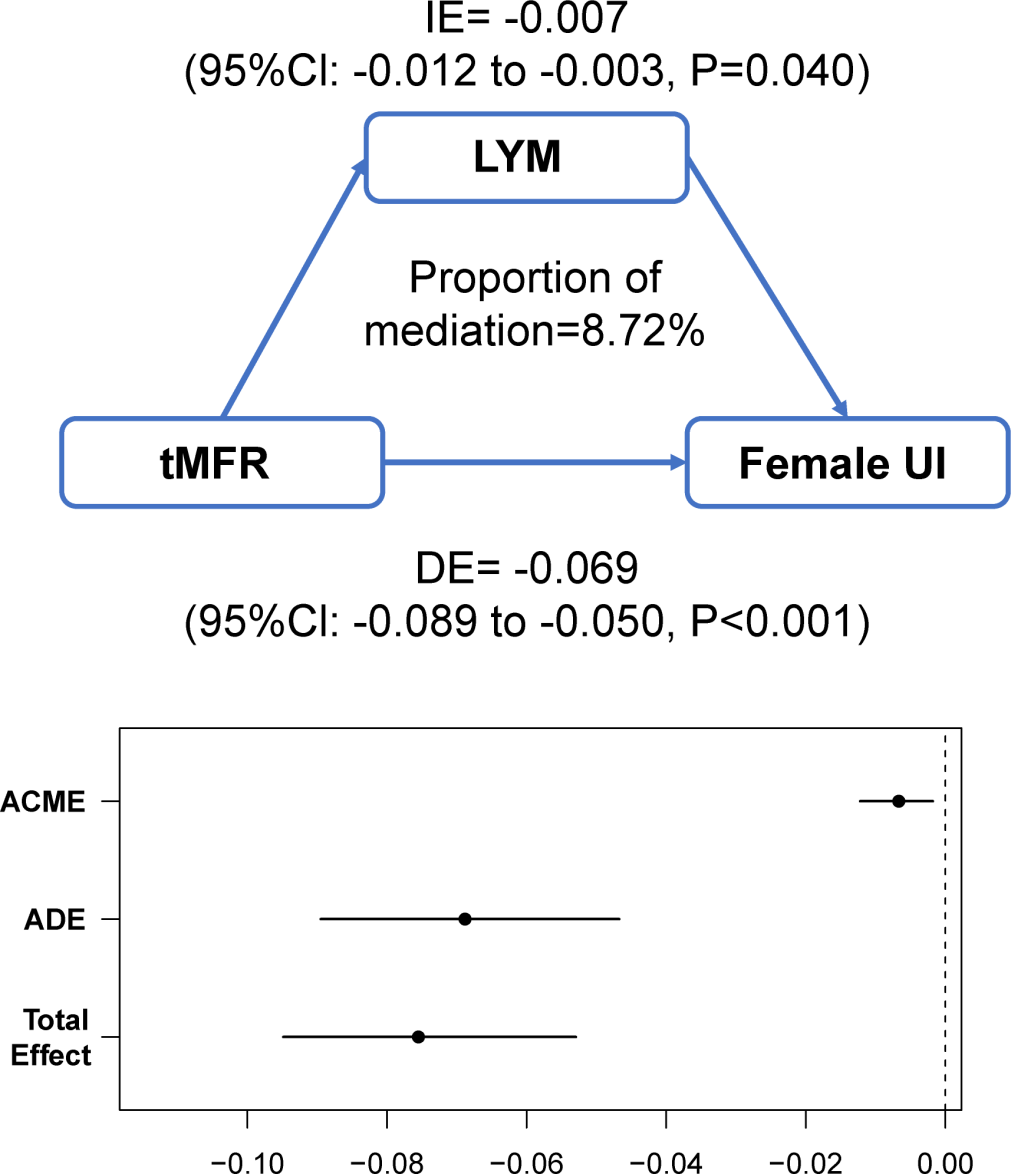
Analysis of the mediating effect between tMFR and female UI. Peripheral blood lymphocytes mediated the association between tMFR and UI in women (IE= -0.007; 95%Cl: -0.012 to -0.003; P=0.040).

The above results lead to the conclusion that tMFR is negatively associated with female UI, so we analyzed the potential mechanism between them by using mediation effects. A community health study found a significant association between C-reactive protein levels and UI in women (20). Additionally, several clinical studies have demonstrated a potential role for inflammation in female overactive bladders (21, 22). It is likely that inflammatory cytokines play a key role in the regulation of connexin expression and the pathogenesis of bladder dysfunction (23, 24). Therefore, blood white blood cells (WBC), the most common indicator of inflammatory conditions, were used to mediate the effect. The mediating variables included blood WBC, lymphocytes (LYM), monocytes (PBMC), neutrophils (GRAN), and eosinophils (E). The results of the mediation analysis are shown in **Figure 5** and **Supplementary Figure 2**. It is concluded that tMFR may affect female UI through the mediating variable LYM (P=0.040).

## Discussion

UI is a prevalent condition in women, significantly impacting their quality of life and mental well-being. In severe cases, it can also elevate the risk of falls and fractures (25, 26). Research has highlighted the close connection between UI and disruptions in homeostasis, making health-related indicators potential predictors of UI risk (27). The tMFR serves as an indirect marker of human health. However, it remains uncertain whether it correlates with female UI within a domestic context and whether it can anticipate the risk of female UI. In light of this uncertainty, the present study conducted a comprehensive analysis using a large sample derived from nationally representative NHANES data. The findings revealed a negative correlation between tMFR and female UI. Moreover, the model constructed based on tMFR demonstrated some predictive efficacy. Consequently, tMFR emerges as a crucial factor in both the prevention of female UI and the enhancement of the quality of life for individuals affected by UI.

Recent research has highlighted that cannabis use heightens the risk of UI in women (28). A potential mechanism behind this association could be that cannabis smoking leads to muscle weakness and fatigue (29), which, in turn, strongly correlates with UI in women. Obesity is recognized as a contributor to the heightened risk of urinary incontinence, particularly when it comes to abdominal obesity. This type of obesity augments the likelihood of urinary incontinence in women by elevating intra-abdominal and bladder pressure (30, 31). The prolonged elevation in intra-abdominal pressure results in varying degrees of damage to the pelvic muscles and nerves, culminating in pelvic floor muscle weakness and dysfunction (32). Consequently, this reduction in support for the pelvic organs transpires (33). Moreover, obese individuals often experience endocrine-metabolic system dysregulation, which fosters the secretion of pro-inflammatory factors within the body (34). This condition subsequently leads to bladder dysfunction (23) and eventually culminates in UI. Weakness or loss of elasticity in the bladder muscles can affect the storage and discharge of urine. Maintaining the health and strength of bladder muscles can reduce the risk of UI. Bladder control is dependent on the function of the muscles. Pelvic floor muscles play a crucial role in preventing UI, as greater muscle mass leads to better urine control. Muscle strength is also closely linked to the nervous system. Impairment of nerve conduction can affect bladder and urethral function. Therefore, maintaining neural health and normal neural conduction is crucial in preventing UI. The principal variable under scrutiny in this paper, tMFR, might amplify the risk of UI in women by virtue of the loss of total body muscle and concurrent increase in fat.

LYM is a type of WBC that play a crucial role in the immune system by fighting infections, clearing pathogens, and maintaining immune balance. Inflammation and infection can cause an increase in LYM, particularly in the urinary tract and bladder regions, which can lead to urinary issues. Abnormal increases in LYM due to urinary inflammation can affect the function of the urethra and bladder, resulting in UI.

This paper’s primary research highlights are centered around the following aspects. Firstly, it presents the inaugural analysis of tMFR as a predictive measure for the risk of UI in women, contributing value to the domain of female UI prevention. Secondly, we identified LYM as a potential mediating variable between tMFR and female UI. Lastly, in comparison to conventional computed tomography and magnetic resonance imaging methods, DXA offers a convenient, non-invasive (35), and cost-effective approach for assessing tMFR in women. Despite the aforementioned advantages, this study also encounters certain limitations. Initially, cross-sectional studies are unable to establish unequivocal causal relationships. Second, the data utilized in this study stem from participants’ self-reported responses, potentially introducing recall and reporting biases. Furthermore, this study solely analyzed correlations using the public NHANES database, which lacks external validation. Prospective studies using clinical data are required to validate the findings.

## Conclusion

Our analysis indicates that tMFR is linked to the risk of UI in women in the United States. Enhancing tMFR may lead to a reduction in the likelihood of UI occurrence. These findings offer fresh insights into UI prevention. Furthermore, the study also unveils the potential involvement of LYM in the pathogenesis of UI among women with low tMFR.

## Data availability statement

The original contributions presented in the study are included in the article/**Supplementary Material**. Further inquiries can be directed to the corresponding authors.

## Ethics statement

We are accountable for all aspects of this study.

## Author contributions

DH: Conceptualization, Data curation, Investigation, Methodology, Software, Supervision, Writing – original draft, Writing – review & editing. HZ: Conceptualization, Data curation, Formal analysis, Methodology, Supervision, Writing – original draft, Writing – review & editing. YY: Data curation, Methodology, Project administration, Validation, Writing – original draft, Writing – review & editing. HQ: Formal analysis, Project administration, Resources, Validation, Writing – original draft, Writing – review & editing. XY: Conceptualization, Data curation, Investigation, Methodology, Project administration, Resources, Software, Writing – original draft. LX: Conceptualization, Funding acquisition, Investigation, Methodology, Project administration, Resources, Software, Supervision, Visualization, Writing – original draft, Writing – review & editing.

## Supplementary material

Supplementary Figure 1 Inclusion criteria for all participants in this paper.

Supplementary Figure 2 WBC, PBMC, GRAN, and E did not mediate the association of tMFR with UI in women (P >0.05).
