# Supplementary figures and images for: Total muscle-to-fat ratio influences urinary incontinence in United States adult women: a population-based study
Source: Front Endocrinol (Lausanne). 2024 Mar 28;15:1309082. doi: 10.3389/fendo.2024.1309082 (PMC11007130; doi:10.3389/fendo.2024.1309082)

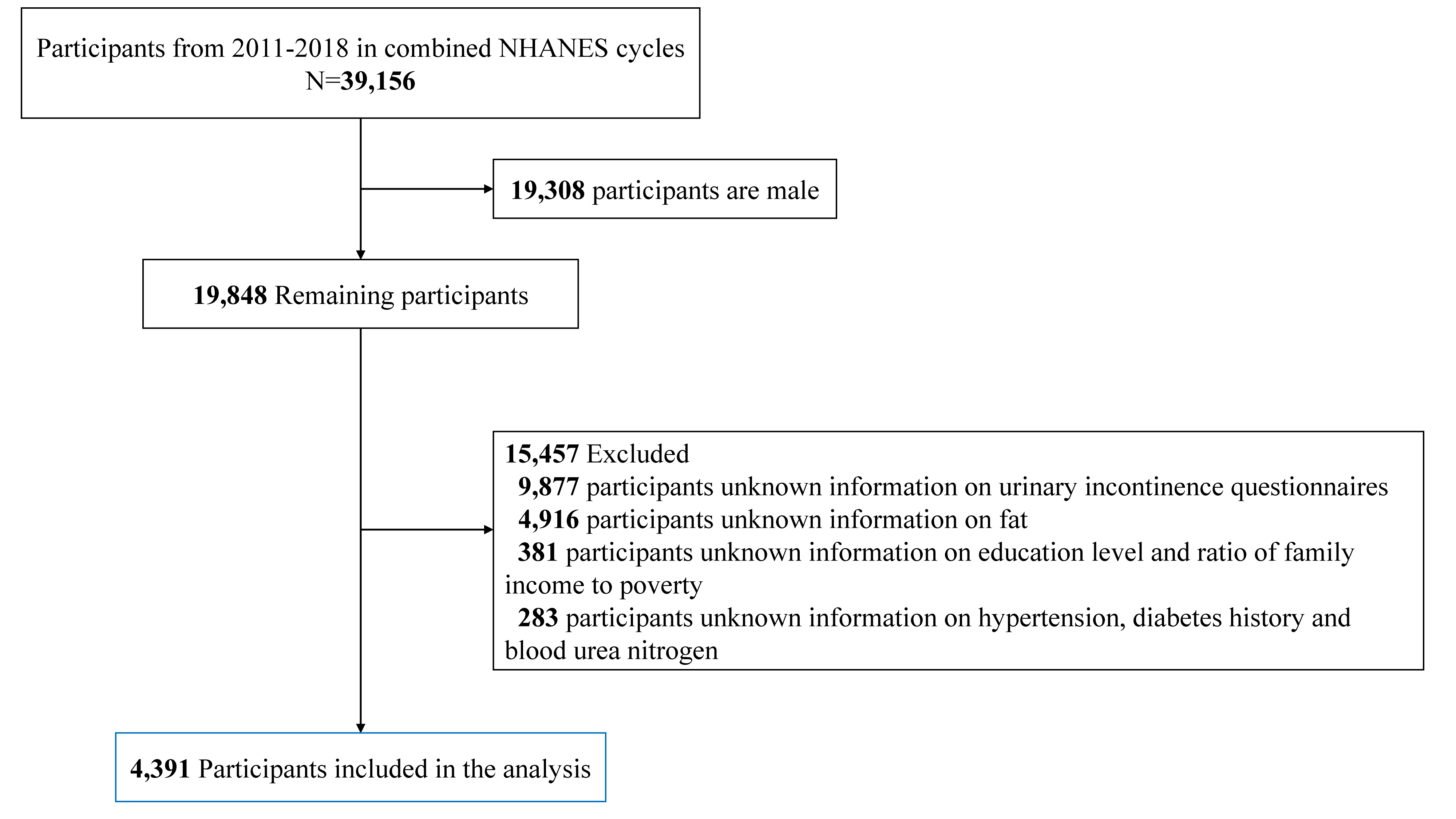

Supplement: Supplementary Figure 1 — Inclusion criteria for all participants in this paper. [file Image_1.tif]

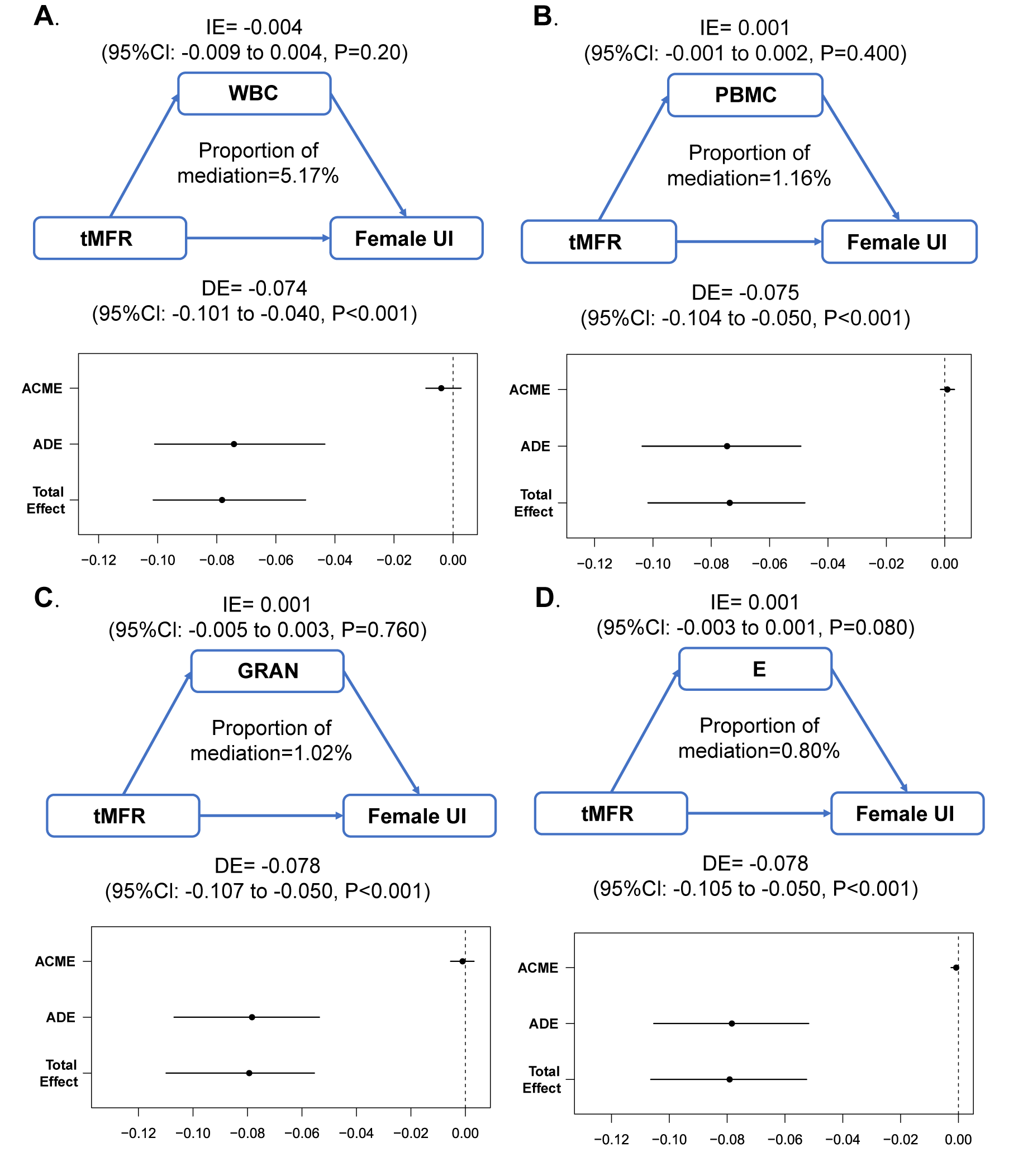

Supplement: Supplementary Figure 2 — WBC, PBMC, GRAN, and E did not mediate the association of tMFR with UI in women (P >0.05). [file Image_2.tif]
