# Supplementary material for: Total muscle-to-fat ratio influences urinary incontinence in United States adult women: a population-based study
Source: Front Endocrinol (Lausanne). 2024 Mar 28;15:1309082. doi: 10.3389/fendo.2024.1309082 (PMC11007130; doi:10.3389/fendo.2024.1309082)
Supplement: Supplementary file 3 [file Table_1.docx]

**Table S1:** Baseline characteristics of NHANES participants between 2011-2018 (n=4391).

| **Characteristic** | **Overall** | **None urinary incontinence** | **Urinary incontinence** | **P value** |
| --- | --- | --- | --- | --- |
| N | 4391 | 3318 (75.6%) | 1073 (24.4%) |  |
| Age | 40.00 [29.50, 49.00] | 38.00 [28.00, 48.00] | 46.00 [34.00, 53.00] | <0.001 |
| Race (%) |  |  |  | <0.001 |
| Mexican American | 650 (14.8) | 483 (14.6) | 167 (15.6) |  |
| Other Hispanic | 468 (10.7) | 363 (10.9) | 105 (9.8) |  |
| Non-Hispanic white | 1641 (37.4) | 1238 (37.3) | 403 (37.6) |  |
| Non-Hispanic black | 891 (20.3) | 629 (19.0) | 262 (24.4) |  |
| Other race | 741 (16.9) | 605 (18.2) | 136 (12.7) |  |
| Education level(%) |  |  |  | <0.001 |
| Less than 9th grade | 209 (4.8) | 156 (4.7) | 53 (4.9) |  |
| 9-11th grade (Includes 12th grade with no diploma) | 446 (10.2) | 322 (9.7) | 124 (11.6) |  |
| High school graduate/GED or equivalent | 858 (19.5) | 610 (18.4) | 248 (23.1) |  |
| Some college or AA degree | 1593 (36.3) | 1200 (36.2) | 393 (36.6) |  |
| College graduate or above | 1285 (29.3) | 1030 (31.0) | 255 (23.8) |  |
| Marital status (%) |  |  |  | <0.001 |
| Married | 2066 (47.1) | 1594 (48.0) | 472 (44.0) |  |
| Widowed | 91 (2.1) | 63 (1.9) | 28 (2.6) |  |
| Divorced | 492 (11.2) | 344 (10.4) | 148 (13.8) |  |
| Separated | 191 (4.3) | 121 (3.6) | 70 (6.5) |  |
| Never married | 1089 (24.8) | 843 (25.4) | 246 (22.9) |  |
| Living with partner | 462 (10.5) | 353 (10.6) | 109 (10.2) |  |
| Ratio of family income to poverty | 2.13 [1.06, 4.08] | 2.22 [1.08, 4.20] | 1.89 [0.95, 3.77] | <0.001 |
| Hypertension (%) |  |  |  | <0.001 |
| Yes | 997 (22.7) | 664 (20.0) | 333 (31.0) |  |
| No | 3394 (77.3) | 2654 (80.0) | 740 (69.0) |  |
| Diabetes (%) |  |  |  | <0.001 |
| Yes | 344 (7.8) | 209 (6.3) | 135 (12.6) |  |
| No | 4047 (92.2) | 3109 (93.7) | 938 (77.4) |  |
| Vigorous recreational activities (%) | |  |  | <0.001 |
| Yes | 1175 (26.8) | 956 (28.8) | 219 (20.4) |  |
| No | 3216 (73.2) | 2362 (71.2) | 854 (79.6) |  |
| Moderate recreational activities (%) | |  |  | 0.002 |
| Yes | 2048 (46.6) | 1592 (48.0) | 456 (42.5) |  |
| No | 2343 (53.4) | 1726 (52.0) | 617 (57.5) |  |
| Blood urea nitrogen (mg/dL) | 11.00 [9.00, 14.00] | 11.00 [9.00, 14.00] | 12.00 [9.00, 14.00] | <0.001 |
| Creatinine (mg/dL) | 0.71 [0.63, 0.81] | 0.71 [0.63, 0.80] | 0.73 [0.64, 0.82] | 0.006 |
| Cotinine (ng/mL) | 0.03 [0.01, 1.35] | 0.03 [0.01, 0.57] | 0.04 [0.01, 22.90] | <0.001 |
| tMFR | 1.46 [1.24, 1.77] | 1.50 [1.28, 1.82] | 1.36 [1.17, 1.61] | <0.001 |
